# Disseminated Infections with *Talaromyces* *marneffei* in Non-AIDS Patients Given Monoclonal Antibodies against CD20 and Kinase Inhibitors

**DOI:** 10.3201/eid2107.150138

**Published:** 2015-07

**Authors:** Jasper F.W. Chan, Thomas S.Y. Chan, Harinder Gill, Frank Y.F. Lam, Nigel J. Trendell-Smith, Siddharth Sridhar, Herman Tse, Susanna K.P. Lau, Ivan F.N. Hung, Kwok-Yung Yuen, Patrick C.Y. Woo

**Affiliations:** The University of Hong Kong, Hong Kong, China

**Keywords:** Talaromyces (Penicillium) marneffei, fungi, CD20, monoclonal antibodies, kinase inhibitors, rituximab, obinutuzumab, ruxolitinib, sorafenib

## Abstract

Clinicians should be aware of these infections, especially in patients from disease-endemic regions.

## Medscape CME Activity

Medscape, LLC is pleased to provide online continuing medical education (CME) for this journal article, allowing clinicians the opportunity to earn CME credit. 

This activity has been planned and implemented in accordance with the Essential Areas and policies of the Accreditation Council for Continuing Medical Education through the joint providership of Medscape, LLC and Emerging Infectious Diseases. Medscape, LLC is accredited by the ACCME to provide continuing medical education for physicians.

Medscape, LLC designates this Journal-based CME activity for a maximum of 1.0 ***AMA PRA Category 1 Credit(s)^TM^***. Physicians should claim only the credit commensurate with the extent of their participation in the activity.

All other clinicians completing this activity will be issued a certificate of participation. To participate in this journal CME activity: (1) review the learning objectives and author disclosures; (2) study the education content; (3) take the post-test with a 75% minimum passing score and complete the evaluation at http://www.medscape.org/journal/eid; (4) view/print certificate.

**Release date: June 15, 2015; Expiration date: June 15, 2016**

## Learning Objectives

Upon completion of this activity, participants will be able to:

1. Distinguish the clinical and epidemiologic characteristics of *T. marneffei* infection, based on a case series report
2. Discuss the recent emergence of disseminated *T. marneffei* infection in non-AIDS patients with hematologic malignant neoplasms treated with targeted therapies
3. Identify possible mechanisms of action underlying disseminated *T. marneffei* infection in non-AIDS patients with hematologic malignant neoplasms treated with targeted therapies

## CME Editor

**Thomas J. Gryczan, MS,** Technical Writer/Editor, *Emerging Infectious Diseases. Disclosure: Thomas J. Gryczan, MS, has disclosed no relevant financial relationships*.

## CME Author

**Laurie Barclay, MD, **freelance writer and reviewer, Medscape, LLC. *Disclosure: Laurie Barclay, MD, has disclosed no relevant financial relationships.*

## Authors

*Disclosures:* ***Harinder Gill, MBBS; Frank Y.F. Lam, MBBS, MRCP(UK), FHKAM; Nigel J. Trendell-Smith, MBBS, FRCPath; Siddharth Sridhar, MBBS, MRCP(UK); Herman Tse, MBBS; Susanna K.P. Lau, MD;*** *and* ***Kwok-Yung Yuen, MD,*** *have disclosed no relevant financial relationships.* ***Jasper F.W. Chan, MBBS, FRCPath, FRCP(Edin),*** *has disclosed the following relevant financial relationships: received travel grants from Pfizer Co. HK Ltd. and Astellas Pharma HK Co. Ltd.* ***Thomas S.Y. Chan, MBBS(Hons), MRCP(UK), FHKAM(Med),*** *has disclosed the following relevant financial relationships: served as a speaker or a member of a speakers bureau for Pfizer.* ***Ivan F.N. Hung, MD,*** *has disclosed the following relevant financial relationships: served as an advisor or consultant for Pfizer (Asia Pacific Capital Advisory Board), MSD; received conference sponsorships from AstraZeneca, Ferring.* ***Patrick C.Y. Woo, MD,*** *has disclosed the following relevant financial relationships: involved in Tigecycline Evaluation Surveillance Trial with Pfizer.*
