# Correction: Vol. 21, No. 7

**DOI:** 10.3201/eid2110.C12110

**Published:** 2015-10

**Keywords:** errata, erratum, correction

An additional case report publication was found after publication of Disseminated Infections with *Talaromyces marneffei* in Non-AIDS Patients Given Monoclonal Antibodies against CD20 and Kinase Inhibitors (J.F.W. Chan al.). An addendum and reference have been added to the article online (http://wwwnc.cdc.gov/eid/article/21/7/15-0138_article).

