# Supplementary material for: Disseminated Infections with Talaromyces marneffei in Non-AIDS Patients Given Monoclonal Antibodies against CD20 and Kinase Inhibitors
Source: Emerg Infect Dis. 2015 Jul;21(7):1101–6. doi: 10.3201/eid2107.150138 (PMC4816330; doi:10.3201/eid2107.150138)
Supplement: Related texts

# Review article

## Abstract

Infections with the fungus *Talaromyces* (formerly *Penicillium*) *marneffei* are rare in patients who do not have AIDS. We report disseminated *T. marneffei* infection in 4 hematology patients without AIDS who received targeted therapy with monoclonal antibodies against CD20 or kinase inhibitors during the past 2 years. Clinicians should be aware of this emerging complication, especially in patients from disease-endemic regions.

*Talaromyces* (formerly *Penicillium*) *marneffei* is a pathogenic, thermal dimorphic fungus that causes systemic mycosis in Southeast Asia. *T. marneffei* infection is characterized by fungal invasion of multiple organ systems, especially blood, bone marrow, skin, lungs, and reticuloendothelial tissues, and is highly fatal, especially when diagnosis and treatment are delayed (*1*,*2*). This disease is found predominantly in AIDS patients and occasionally those with cell-mediated immunodeficiencies involving the interleukin-12/interferon-γ (IFN-γ) signaling pathway, such as congenital STAT1 mutations or acquired autoantibodies against IFN-γ (*1*,*3*–*6*). The infection has rarely been reported among hematology patients, including those from disease-endemic regions (*7*,*8*). 

At Queen Mary Hospital in Hong Kong, a 1,600-bed university teaching hospital that has a hematopoietic stem cell transplantation service, where a wide range of invasive fungal infections have been observed (*9*,*10*), only 3 cases of *T. marneffei* infection were encountered in >2,000 hematology patients in the past 20 years, despite the long-standing availability of mycologic culture and serologic testing (*7*,*8*,*11*,*12*). In contrast, the infection was commonly reported among AIDS patients (*13*).

In the past 2 years, we have been alerted by 4 unprecedented cases of disseminated *T. marneffei* infection among non-AIDS hematology patients given targeted therapies, including monoclonal antibodies (mAbs) against CD20 and kinase inhibitors, which are being increasingly used in recent years. We report details for these 4 hematology case-patients. The study was approved by the institutional review board of The University of Hong Kong/Hospital Authority Hong Kong West Cluster in Hong Kong.

## Case-Patient 1

Patient 1 was a 56-year-old Filipino man with Waldenström macroglobulinemia, idiopathic thrombocytopenic purpura, and primary biliary cirrhosis. He had fever, night sweating, productive cough, and left facial pain for 1 week and bloody diarrhea for 2 days. He had previously received fludarabine, dexamethasone, and rituximab (mAb against CD20, 18 months earlier) for treatment of Waldenström macroglobulinemia (Table 1). The idiopathic thrombocytopenic purpura was controlled with intravenous immunoglobulin and maintenance prednisolone and mycophenolate sodium. A chest radiograph showed a small cavitary lesion in the right lower lobe. His symptoms and signs did not resolve after he received empirical intravenous imipenem/cilastatin and metronidazole (Table 2).

**Table 1:** Characteristics of 4 case-patients with disseminated *Talaromyces marneffei* infection after targeted therapies*

Characteristic	Case-patient 1	Case-patient 2	Case-patient 3	Case-patient 4
Age, y/sex	56/M	44/M	63/M	67/M
Concurrent conditions	Waldenström macroglobulinemia, idiopathic thrombocytopenic purpura, primary biliary cirrhosis	Chronic lymphocytic leukemia	Myelofibrosis with splenectomy, diabetes mellitus	Acute myeloid leukemia, hypertension
Targeted therapy	Rituximab	Rituximab and obinutuzumab	Ruxolitinib	Sorafenib
Action of therapy	mAb against CD20	mAb against CD20	JAK-1/2 inhibitor	Multikinase inhibitor
Time interval, mo†	18	14 (rituximab) and concomitant (obinutuzumab)	Concomitant	Concomitant
Cumulative dose before *T. marneffei* infection	700 mg/dose iv x 4 doses	700 mg/dose IV x 13 doses (rituximab) and 1,000 mg IV x 3 doses (obinutuzumab)	10–20 mg 2×/d oral x 6.5 mo	400 mg 2×/d oral x 8 mo
Other immunosuppressants (time interval, mo)†	Fludarabine and dexamethasone (39), prednisolone 10 mg/d and mycophenolate sodium 360 mg 2×/d (concomitant)	Fludarabine and cyclophosphamide (48), CHOP (36), bendamustine (13)	None	Mitoxantrone and etoposide (21), daunarubicin (20), clofarabine (18), azacitidine (15), decitabine (15), cytarabine (14)
Clinical manifestations	Terminal ileitis, cerebral abscesses, nasopharyngitis, and multiple cavitary lung lesions	Marrow infiltration and fungemia	Right cervical lymphadenitis and multiple cavitary lung lesions	Fungemia
Specimens positive for *T. marneffei*	Feces, and terminal ileal and nasopharyngeal biopsy specimens	Blood and bone marrow aspirate	Right cervical lymph node	Blood
Highest serum antibody titer against *T. marneffei*	1:320	<1:40	1:320	<1:40
Antifungal treatment (duration, mo)	Amphotericin B (2 weeks) and voriconazole (>21)	Amphotericin B (2 weeks) and itraconaozle (5)	Amphotericin B (2 weeks) and voriconazole (>6)	Amphotericin B (2 weeks) and voriconazole (>5)
Other opportunistic infections	None	Bacteremia (*Mycobacterium chelonae*, *Enterococcus faecium*, and MRCNS), fungemia (*Candida glabrata*), HSV oral mucositis, PJP	Bacteremia (*Klebsiella pneumoniae*)	Herpes zoster at right occiput
Clinical outcome	Responded to antifungal treatment	Clearance of *T. marneffei* fungemia but died of MODS and multiple infections 5 mo after *T. marneffei* infection	Responded to antifungal treatment	Responded to antifungal treatment

*mAb, monoclonal antibody; JAK, Janus kinase; IV, intravenous; CHOP, cyclophosphamide, hydroxydaunorubicin, oncovin, and prednisolone; MRCNS, methicillin-resistant coagulase-negative *Staphylococcus*; HSV, herpes simplex virus; PJP, *Pneumocystis jiroveci* pneumonia; MODS, multiple organ dysfunction syndrome. †Time interval between end of therapy and onset of symptoms for *T. marneffei* infection.

**Table 2:** Laboratory results for 4 case-patients with disseminated *Talaromyces marneffei* infection after targeted therapies*

Laboratory parameter	Case-patient 1	Case-patient 2	Case-patient 3	Case-patient 4
Hematologic†				
Leukocytes, x 10^9^ cells/L	12.08	0.91	4.93	33.79
Neutrophils, x 10^9^ cells/L	11.01	0.45	3.11	8.45 (with blasts)
Lymphocytes, x 10^9^ cells/L	0.83 (CD4+: 315/µL)‡	0.45	1.05	9.12 (with blasts)
Hemoglobin, g/dL	12.3	10.3	8.0	9.2
Platelets, x 10^9^/L	250	5	539	15
Biochemical†				
Sodium, mmol/L	136	135	139	138
Potassium, mmol/L	3.5	4.1	3.7	4.4
Creatinine, µmol/L	101	111	78	92
Albumin, g/L	40	32	39	37
Globulin, g/L	34	36	36	39
Total bilirubin, µmol/L	8	9	13	19
ALP, U/L	234	163	112	96
ALT, U/L	79	20	32	61
AST, U/L	38	9	28	123
LDH, U/L	209	97	352	2,069
Immunologic				
Combined HIV antibody/antigen	Negative	Negative	Negative	Negative
Autoantibody against IFN-γ	Negative	Negative	Negative	Negative
Microbiologic				
Blood culture	No bacteria and fungi	*T. marneffei; Mycobacterium chelonae*, *Enterococcus faecium*, MRCNS, and *Candida glabrata*§	*Klebsiella pneumoniae*§	*T. marneffei*
Bone marrow aspirate	ND	*T. marrneffei*	ND	ND
Sputum culture	Negative for pathogenic bacteria, AFB, and fungi	Negative for pathogenic bacteria, AFB, and fungi	Negative for pathogenic bacteria, AFB, and fungi	Negative for pathogenic bacteria, AFB, and fungi
Urine culture	No bacteria and fungi	No bacteria and fungi	No bacteria and fungi	No bacteria and fungi
Stool culture	*T. marneffei*; negative for pathogenic bacteria, including *Clostridium difficile* and AFB	ND	ND	ND
Serum CMV pp65 antigen	Negative	Negative	Negative	Negative
Other	Stool for *C. difficile* toxin (negative); serum for *Entamoeba histolytica* antibody (negative); multiple blood smears for *Plasmodium* sp. (negative)	BAL: *Pneumocystis jiroveci* (smear-positive)	Cervical lymph node: *T. marneffei* (culture-positive)	

*ALP, alkaline phosphatase; ALT, alanine aminotransferase; AST, aspartate aminotransferase; LDH, lactate dehydrogenase; IFN-γ, interferon-γ; MRCNS, methicillin-resistant coagulase-negative *Staphylococcus*; ND, not done; AFB, acid-fast bacilli; CMV, cytomegalovirus; BAL, bronchoalveolar lavage. Reference ranges: leukocytes; 3.89–9.93 × 10^9^ cells/L; neutrophils, 2.01–7.42 × 10^9^ cells/L; lymphocytes, 1.06–3.61 × 10^9^ cells/L; hemoglobin, 13.3–17.7g/dL; platelets, 162–341 × 10^9^/L; sodium, 136–148 mmol/L; potassium, 3.6–5.0 mmol/L; creatinine, 67–109 µmol/L; albumin, 39–50 g/L; globulin, 24–37 g/L; total bilirubin, 4–23 µmol/L; ALP, 42–110 U/L; ALT, 8–58 U/L; AST, 5–38 U/L; LDH, 118–221 U/L. †Results at presentation. ‡Reference range of CD4+ lymphocyte count: 415–1,418 cells/µL. §Bacteremia caused by *M. chelonae, E. faecium,* MRCNS, and candidemia in case-patient 2, and bacteremia caused by *K. pneumoniae* in case-patient 3 occurred after recovery from *T. marneffei* infection and prolonged hospitalization.

A colonoscopy showed multiple shallow ulcers at the terminal ileum (Figure 1). Histologic analysis of an ulcer biopsy specimen showed slough of an acutely inflamed ulcer but no microorganisms. However, histologic analysis of a specimen from a nasopharyngeal biopsy performed for persistent left facial pain showed abundant yeast cells engulfed by foamy macrophages (Figure 2). Culture of terminal ileal ulcer biopsy specimens, stool samples, and nasopharyngeal biopsy specimens yielded *T. marneffei*. A contrast-enhanced cranial computed tomography (CT) scan showed 2 lesions (3–4-mm) with rim enhancement and perifocal edema at the right occipital and left parieto-occipital lobes. A thoracic CT scan showed 2 cavitary lesions (4–8 mm) in the right upper and lower lobes.

**Figure 1:** Multiple, shallow, oozing ulcers at the terminal ileum (arrows) detected by colonoscopy on day 4 of hospitalization for case-patient 1, who had a disseminated infection with *Talaromyces marneffei*.

**Figure 2:** Nasopharyngeal biopsy specimen from case-patient 1, who had a disseminated infection with *Talaromyces marneffei*. A) Grocott silver staining showing abundant yeast cells (arrows) with central septa 4–5 µm in diameter. B) Hematoxylin and eosin staining showing necrotic material admixed with blood and fibrin with aggregates of foamy macrophages (arrow). Scale bars indicate 5 μm.

Immunologic testing showed that the patient was negative for HIV and autoantibodies against IFN-γ. His CD3+ and CD8+ counts were within references ranges, but he had mild CD4+ lymphopenia (Table 2). His fever and symptoms resolved with after 2 weeks of treatment with intravenous liposomal amphotericin B, followed by oral voriconazole. Reassessment colonoscopy (at 2 months) and CT scan (at 6 months) showed complete resolution of all lesions.

## Case-Patient 2

Patient 2 was a 44-year-old Chinese man who had fever for 2 days. He had previously received chemotherapy and mAbs against CD20 (rituximab, 14 months earlier; obinutuzumab, concomitant) for refractory chronic lymphocytic leukemia (CLL) involving bone marrow (Table 1). He was empirically given intravenous piperacillin/tazobactam and anidulafungin (Table 2). Histologic analysis of a trephine biopsy specimen showed persistent CLL with plasmacytic differentiation, and Grocott staining showed yeasts with central septa in small clusters. Culture of peripheral blood and bone marrow aspirate yielded *T. marneffei*. A change in antifungal treatment to intravenous amphotericin B led to defervescence and clearance of fungemia. He was given oral itraconazole as maintenance therapy. He remained well until 2 months later when he was hospitalized for deteriorating CLL complicated by neutropenic fever with multiorgan failure caused by other opportunistic infections (Table 1). He died 5 months after the episode of disseminated *T. marneffei* infection.

## Case-Patient 3

Patient 3 was a 63-year-old Chinese man with myelofibrosis and well-controlled diabetes mellitus. He had intermittent fever, right cervical lymphadenopathy, and productive cough for 4 months. He was given ruxolitinib (kinase inhibitor) 6 months before symptom onset because of transfusion-dependent myelofibrosis despite splenectomy 4 years earlier (Table 1). A chest radiograph and thoracic CT scan showed multiple cavitary lesions and consolidation. Bronchoalveolar lavage was negative for bacteria, fungi, and mycobacteria. A serum cryptococcal antigen test result was negative. He was empirically given intravenous imipenem/cilastatin and oral doxycycline, but his symptoms persisted. A right cervical lymph node culture yielded *T. marneffei*. His symptoms and radiologic abnormalities resolved after treatment with intravenous amphotericin B for 2 weeks, followed by oral voriconazole for 6 months.

## Case-Patient 4

Patient 4 was a 67-year-old Chinese man with acute myeloid leukemia and hypertension. He had fever and malaise for 2 days without localizing signs. He had been given sorafenib (kinase inhibitor) 8 months earlier for chemotherapy-refractory acute myeloid leukemia (Table 1). His fever did not respond to intravenous meropenem. Subsequently, 2 sets of blood cultures yielded *T. marneffei*. He was given intravenous amphotericin B for 2 weeks, followed by oral voriconazole. He remained well at follow-up 6 months after symptom onset.

## Discussion

*T. marneffei* infection is an emerging complication in hematology patients receiving targeted therapies. Historically, *T. marneffei* infection has rarely been seen in non-AIDS patients, even in disease-endemic regions. During 1994–2014, only 3 other cases were observed in our hematology patients (*7*,*8*,*11*). None of 47 patients with *T. marneffei* infection in another large local case series during 1994–2004 had hematologic disease (*13*). In the past 20 years, there has been no change in methods for laboratory diagnosis of *T. marneffei* infection or a marked increase in the number of hematology patients in our hospital. Therefore, these 4 cases indicate an increase in the incidence of *T. marneffei* infection in these patients. Although other immunosuppressants given to case-patients 1, 2, and 4 might have contributed to overall immunosuppression, none of these immunosuppressants, which have been used for years, have been associated with *T. marneffei* infection. Because use of targeted therapies is increasing in diverse patient groups, clinicians should be aware of this emerging complication, especially in patients from disease-endemic regions who have received these therapies with other immunosuppressants.

The exact mechanisms through which these targeted therapies lead to *T. marneffei* infection remain incompletely understood. Rituximab and obinutuzumab (used by case-patients cases 1 and 2) are mAbs against CD20 that predominantly target B cells. Unlike T cells, the role of B cell–mediated humoral response in *T. marneffei* infection is poorly defined. Although case-patient 1 had mild CD4+ lymphopenia probably related to concomitant use of prednisolone and mycophenolate sodium, *T. marneffei* infection is rarely seen in patients with CD4+ counts >300/µL (*1*). We postulate that B cell dysfunction might have impaired production of neutralizing antibodies against key virulence factors of *T. marneffei* or might involve impairment of cytokine-producing B cells, which are essential for T helper cell function (*14*). 

More severe infections with fungemeia and bone marrow involvement developed in case-patients 2 and 4, who had undetectable levels of serum antibodies against *T. marneffei*. Correspondingly, case-patients 1 and 3, who had antibody titers >1:320, did not have positive blood culture results (Table 2). Symptoms developed in case-patient 1 more than a 1 year after he completed therapy with mAbs against CD20. This finding might be related to long-lasting B cell–depleting effects of mAbs against CD20 (*15*).

Regarding kinase inhibitors (used by cases-patients 3 and 4), ruxolitinib is a selective Janus kinase (JAK)-1/2 inhibitor that prevents signal transduction for type I/II cytokines, including IFN-γ, by interfering with the JAK-STAT signaling pathway. Use of ruxolitinib has been associated with opportunistic infections caused by intracellular pathogens, such as *Mycobacterium tuberculsosis* and *Cryptococcus neoformans* (*16*,*17*). Similarly, patients with impaired JAK-STAT signaling, but not those with diabetes mellitus or splenectomy (case-patient 3), are predisposed to *T. marneffei* infection (*6*). Sorafenib is a multikinase inhibitor with various immunomodulatory effects, including impaired T-cell response and proliferation and reduced IFN-γ production (*18*). These immune defects have been associated with reactivation of latent tuberculosis and might also predispose patients to opportunistic infections caused by intracellular organisms such as *T. marneffei* (*18*).

 The recognition of disseminated *T. marneffei* infection as an emerging complication in non-AIDS patients treated with targeted therapy has major public health implications. In regions to which *T. marneffei* infection is endemic, serologic surveillance for patients receiving targeted therapy might be useful in the early diagnosis of *T. marneffei* infection, as in the case of AIDS patients (*19*). In non-endemic regions, such as the United States, clinicians should be vigilant of this infrequent infection in at-risk hematology patients who have resided in or are returning from disease-endemic areas.

## References

[1] Vanittanakom N, Cooper CR Jr, Fisher MC, Sirisanthana T. *Penicillium marneffei* infection and recent advances in the epidemiology and molecular biology aspects. Clin Microbiol Rev. 2006;19:95–110. 10.1128/CMR.19.1.95-110.200616418525PMC1360277

[2] Samson RA, Yilmaz N, Houbraken J, Spierenburg H, Seifert KA, Peterson SW, Phylogeny and nomenclature of the genus *Talaromyces* and taxa accomodated in *Penicillium* subgenus *Biverticillium.* Stud Mycol. 2011;70:159–83. 10.3114/sim.2011.70.0422308048PMC3233910

[3] Tang BS, Chan JF, Chen M, Tsang OT, Mok MY, Lai RW, Disseminated penicilliosis, recurrent bacteremic nontyphoidal salmonellosis, and burkholderiosis associated with acquired immunodeficiency due to autoantibody against gamma interferon. Clin Vaccine Immunol. 2010;17:1132–8. 10.1128/CVI.00053-1020445006PMC2897261

[4] Chan JF, Trendell-Smith NJ, Chan JC, Hung IF, Tang BS, Cheng VC, Reactive and infective dermatoses associated with adult-onset immunodeficiency due to anti-interferon-gamma autoantibody: Sweet’s syndrome and beyond. Dermatology. 2013;226:157–66. 10.1159/00034711223652167

[5] Lee PP, Chan KW, Lee TL, Ho MH, Chen XY, Li CH, Penicilliosis in children without HIV infection – are they immunodeficient? Clin Infect Dis. 2012;54:e8–19. 10.1093/cid/cir75422065867

[6] Lee PP, Mao H, Yang W, Chan KW, Ho MH, Lee TL, *Penicillium marneffei* infection and impaired IFN-γ immunity in humans with autosomal-dominant gain-of-phosphorylation STAT1 mutations. J Allergy Clin Immunol. 2014;133:8948–6.e5. 10.1016/j.jaci.2013.08.05124188975

[7] Wong SS, Woo PC, Yuen KY. *Candida tropicalis* and *Penicillium marneffei* mixed fungaemia in a patient with Waldenstrom’s macroglobulinaemia. Eur J Clin Microbiol Infect Dis. 2001;20:132–5. 10.1007/PL0001124311305468

[8] Woo PC, Lau SK, Lau CC, Chong KT, Hui WT, Wong SS, *Penicillium marneffei* fungaemia in an allogeneic bone marrow transplant recipient. Bone Marrow Transplant. 2005;35:831–3. 10.1038/sj.bmt.170489515765113

[9] Cheng VC, Chan JF, Ngan AH, To KK, Leung SY, Tsoi HW, Outbreak of intestinal infection due to *Rhizopus microsporus.* J Clin Microbiol. 2009;47:2834–43. 10.1128/JCM.00908-0919641069PMC2738128

[10] Yuen KY, Woo PC, Ip MS, Liang RH, Chiu EK, Siau H, Stage-specific manifestation of infection and impaired mold infections in bone marrow transplant recipients: risk factors and clinical significance of positive concentrated smears. Clin Infect Dis. 1997;25:37–42. 10.1086/5144929243031

[11] Wong SS, Wong KH, Hui WT, Lee SS, Lo JY, Cao L, Differences in clinical and laboratory diagnostic characteristics of penicilliosis marneffei in human immunodeficiency virus (HIV)- and non-HIV-infected patients. J Clin Microbiol. 2001;39:4535–40. 10.1128/JCM.39.12.4535-4540.200111724878PMC88582

[12] Yuen KY, Wong SS, Tsang DN, Chau PY. Serodiagnosis of *Penicillium marneffei* infection. Lancet. 1994;344:444–5. 10.1016/S0140-6736(94)91771-X7914566

[13] Wu TC, Chan JW, Ng CK, Tsang DN, Lee MP, Li PC. Clinical presentations and outcomes of *Penicillium marneffei* infections: a series from 1994 to 2004. Hong Kong Med J. 2008;14:103–9 .18382016

[14] Dang VD, Hilgenberg E, Ries S, Shen P, Fillatreau S. From the regulatory functions of B cells to the identification of cytokine-producing plasma cell subsets. Curr Opin Immunol. 2014;28:77–83. 10.1016/j.coi.2014.02.00924637161

[15] Anolik JH, Friedberg JW, Zheng B, Barnard J, Owen T, Cushing E, B cell reconstitution after rituximab treatment of lymphoma recapitulates B cell ontogeny. Clin Immunol. 2007;122:139–45. 10.1016/j.clim.2006.08.00917008130

[16] Wysham NG, Sullivan DR, Allada G. An opportunistic infection associated with ruxolitinib, a novel janus kinase 1,2 inhibitor. Chest. 2013;143:1478–9. 10.1378/chest.12-160423648912PMC5991580

[17] Hopman RK, Lawrence SJ, Oh ST. Disseminated tuberculosis associated with ruxolitinib. Leukemia. 2014;28:1750–1. 10.1038/leu.2014.10424625550

[18] Teo M, O’Connor TM, O’Reilly SP, Power DG. Sorafenib-induced tuberculosis reactivation. Onkologie. 2012;35:514–6. 10.1159/00034182923007150

[19] Wang YF, Xu HF, Han ZG, Zeng L, Liang CY, Chen XJ, Serological surveillance for Penicillium marneffei infection in HIV-infected patients during 2004–2011 in Guangzhou, China. Clin Microbiol Infect. 2014;Dec 26:pii:S1198-743X(14)00167-0. Epub ahead of print.10.1016/j.cmi.2014.12.01425677258

# Review article

## Medscape CME Quiz

## Earning CME Credit

To obtain credit, you should first read the journal article. After reading the article, you should be able to answer the following, related, multiple-choice questions. To complete the questions (with a minimum 75% passing score) and earn continuing medical education (CME) credit, please go to http://www.medscape.org/journal/eid. Credit cannot be obtained for tests completed on paper, although you may use the worksheet below to keep a record of your answers. You must be a registered user on Medscape.org. If you are not registered on Medscape.org, please click on the “Register” link on the right hand side of the website to register. Only one answer is correct for each question. Once you successfully answer all post-test questions you will be able to view and/or print your certificate. For questions regarding the content of this activity, contact the accredited provider, CME@medscape.net. For technical assistance, contact CME@webmd.net. American Medical Association’s Physician’s Recognition Award (AMA PRA) credits are accepted in the US as evidence of participation in CME activities. For further information on this award, please refer to http://www.ama-assn.org/ama/pub/about-ama/awards/ama-physicians-recognition-award.page. The AMA has determined that physicians not licensed in the US who participate in this CME activity are eligible for *AMA PRA Category 1 Credits™*. Through agreements that the AMA has made with agencies in some countries, AMA PRA credit may be acceptable as evidence of participation in CME activities. If you are not licensed in the US, please complete the questions online, print the certificate and present it to your national medical association for review.

## Article Title:  Disseminated Infections with *Talaromyces* *marneffei* in Non-AIDS Patients Given Monoclonal Antibodies against CD20 and Kinase Inhibitors

## CME Questions

**1. According to the case series report by Chan and colleagues, which of the following statements about the clinical and epidemiologic characteristics of *Talaromyces marneffei* infection is correct? **A. *T. marneffei* infection is the most important pathogenic thermal dimorphic fungus causing systemic mycosis in Southeast AsiaB. *T. marneffei* infection is typically limited to the gastrointestinal and/or urinary tractC. *T. marneffei* infection is usually self-limited with a good prognosis for recoveryD. *T. marneffei* infection is common among non-AIDS hematology patients**2. Your patient is a 57-year-old Chinese man with acute myeloid leukemia and fever. According to the case series report by Chan and colleagues, which of the following statements about the recent emergence of disseminated *T. marneffei* infection in non-AIDS patients with hematologic malignant neoplasms treated with targeted therapies is correct?**A. The appearance of 4 cases in the past 2 years is the result of a change in the methodologies of a laboratory diagnosis of *T. marneffei* infectionB. The appearance of 4 cases in the past 2 years is the result of a dramatic increase in the number of hematology patients at the investigators' hospitalC. Recent emergence of disseminated *T. marneffei* infection is most likely because of overall immunosuppressionD. Recent emergence of disseminated *T. marneffei* infection is most likely because of targeted therapies, such as anti-CD20 monoclonal antibodies and kinase inhibitors**3. According to the case series report by Chan and colleagues, which of the following statements about possible mechanisms of action underlying disseminated *T. marneffei* infection in non-AIDS patients with hematologic malignant neoplasms treated with targeted therapies would most likely be accurate? **A. Rituximab and obinutuzumab used in 2 cases are anti-CD20 monoclonal antibodies that predominantly target T cellsB. *T. marneffei* infection is usually seen at CD4+ counts of more than 300/µLC. Patients with B-cell dysfunction may have impaired production of neutralizing antibodies against key virulence factors of *T. marneffei* D. The investigators do not suggest any role of cytokine-producing B-cells

## Activity Evaluation

**1:** The activity supported the learning objectives.

Strongly Disagree				Strongly Agree
1	2	3	4	5
**2. The material was organized clearly for learning to occur.**				
Strongly Disagree				Strongly Agree
1	2	3	4	5
**3. The content learned from this activity will impact my practice.**				
Strongly Disagree				Strongly Agree
1	2	3	4	5
**4. The activity was presented objectively and free of commercial bias.**				
Strongly Disagree				Strongly Agree
1	2	3	4	5
